# Predictors of First-Line Progression-Free Survival in Testicular Germ Cell Tumors: A Retrospective Cohort Study

**DOI:** 10.3390/cancers18152419

**Published:** 2026-07-27

**Authors:** Andreea Parosanu, Cornelia Nitipir, Cristian Iaciu, Miruna Stanciu, Ioanel Sinescu, Cătălin Baston

**Affiliations:** 1 Faculty of Medicine, Carol Davila University of Medicine and Pharmacy, 8 Sanitary Heroes Boulevard, 050474 Bucharest, Romania; andreea.parosanu@umfcd.ro (A.P.); catalin.baston@umfcd.ro (C.B.); 2 Elias University Emergency Hospital, 011461 Bucharest, Romania; 3 Prof. Dr. Agrippa Ionescu Clinical Emergency Hospital, 077015 Balotești, Romania; 4 Fundeni Clinical Institute, 022328 Bucharest, Romania

**Keywords:** testicular germ cell tumors, relapse, prognostic factors

## Abstract

**Background**: Testicular germ cell tumors are usually highly responsive to platinum-based treatment. However, outcomes remain heterogeneous. This exploratory retrospective study assessed factors associated with first-line progression-free survival and with the need for second-line systemic therapy. **Methods**: We reviewed 41 patients treated for testicular germ cell tumors at a single oncology center. Clinical, pathological, metastatic, treatment-related, and biochemical variables were extracted from medical records. First-line progression-free survival was evaluated using exploratory univariable Cox regression. Variables associated with second-line treatment were assessed using exploratory univariable analyses. **Results**: The median age was 37 years and most of the patients had non-seminomatous histology. A greater mean decline of initially elevated serum markers after the first chemotherapy cycle was associated with longer first-line progression-free survival in both the overall cohort and the non-seminomatous subgroup. The need for second-line systemic treatment was associated with stage III disease, metastasis at diagnosis, and elevated baseline serum tumor markers. **Conclusions**: In this cohort, baseline disease burden and marker elevation were the clearest adverse clinical signals. Moreover, early marker decline may add information about treatment sensitivity and tailor follow-up.

## Simple Summary

Germ cell tumors are uncommon malignancies that mainly occur in adolescents and young men. The primary tumor may arise in the testis or at extragonadal sites, most commonly in the mediastinum or retroperitoneum. Because of their marked sensitivity to platinum-based chemotherapy, most patients can be cured. Nevertheless, a minority experience disease recurrence after initial treatment and require further systemic therapy. This retrospective, single-center study explored potential predictors of first-line progression-free survival. The analysis indicated that disease extent, baseline serum tumor marker levels, treatment delivery, and early biochemical changes after the first chemotherapy cycle may influence clinical outcomes and help identify patients at greater risk of progression.

## 1. Introduction

Germ cell tumors comprise a heterogeneous group of tumors originating from primordial germ cells. Most germ cell tumors arise in the gonads, while about 5% occur at extragonadal sites, probably because primordial germ cells migrate abnormally during embryonic development. The anterior mediastinum is the most common extragonadal location, representing more than half of cases, followed by the retroperitoneum, which accounts for approximately 40%. Exceptionally rare primary sites include the pineal and suprasellar regions, as well as the sacrococcygeal area [1,2,3,4,5].

Although testicular cancer accounts for only approximately 2% of all malignancies diagnosed in men, it is the most common solid tumor among adolescents and young adult males. Overall, approximately 1 in 250 men will develop testicular cancer during their lifetime [6]. It has a high probability of cure when diagnosis, staging, and treatment are delivered promptly. Across contemporary series, overall cure rates exceed 90%, reflecting the exceptional chemosensitivity of germ cell tumors [7,8]. Despite this favorable background, testicular cancer is not a single prognostic entity. Outcomes differ according to histological subtype, anatomical stage, metastatic pattern, and serum tumor marker profile. Most patients present with stage I disease and have excellent long-term outcomes, but stage III disease reflects metastatic involvement, since stage IV is not used in the conventional staging. Even among patients with distant metastases, cure remains possible, particularly when risk-adapted platinum chemotherapy is delivered according to established guidelines [9,10,11,12,13].

The introduction of cisplatin-based chemotherapy transformed the management of advanced germ cell tumors and remains central to both first-line and salvage treatment. Regimens such as BEP, EP, VIP, and TIP are used according to stage and risk group. However, some patients experience early progression, incomplete response, relapse, or the need for second-line treatment [14,15,16,17,18]. For this reason, real-world data on factors associated with first-line progression-free survival remain clinically useful. Baseline tumor markers, metastatic status, treatment delivery, and early marker kinetics are particularly relevant because they are readily available in routine practice and may help identify patients who need more intensive monitoring.

The present retrospective study aims to evaluate clinical, pathological, treatment-related, and biochemical variables associated with first-line progression-free survival in patients with testicular germ cell tumors.

## 2. Materials and Methods

This was a retrospective, single-center cohort study including 41 patients diagnosed with testicular germ cell tumors and treated at Elias University Emergency Hospital between 2017 and 2026. Patients with seminomatous or non-seminomatous germ cell tumors and available clinical, pathological, treatment, and follow-up data were included. Clinical and pathological data were collected from medical records. Variables included age, risk factors, primary tumor location, histology, disease stage, metastatic status, serum tumor markers, first-line treatment, treatment timing, progression-free survival, need for second-line treatment, and survival status.

Tumor stage was assigned according to the TNM classification. For patients with advanced germ cell tumors, the IGCCCG prognostic group was determined retrospectively. Patients were classified as having good-, intermediate-, or poor-risk disease. The classification was considered unavailable when baseline data were incomplete.

Overall survival was defined as the time from diagnosis to death from any cause. Patients who were alive at the time of analysis were censored at the date of their last follow-up. First-line progression-free survival was defined as the time from initiation of first-line systemic therapy to documented radiological or biochemical progression, initiation of second-line systemic treatment, death, or last follow-up, whichever occurred first. Patients without progression or subsequent systemic therapy were censored at the date of last follow-up. Treatment postponement greater than 7 days during systemic therapy was defined as a delay exceeding 7 days in the administration of a planned chemotherapy cycle or treatment sequence, most commonly due to treatment-related adverse events such as anemia or neutropenia.

Early tumor marker response was assessed retrospectively by calculating the percentage change in AFP, β-hCG, and LDH after the first chemotherapy cycle. When multiple markers were elevated at baseline, the mean percentage decline across these markers was calculated. Marker decline was analyzed primarily as a continuous variable. A post hoc exploratory cutoff of ≥80% was also used to identify patients with a pronounced biochemical response. This threshold was selected for descriptive purposes and was not derived from the validated GETUG kinetic model, which was not formally applied in this study.

This study was approved by the Ethics Committee of Elias University Emergency Hospital (approval number 13052026-1; date of approval: 13 May 2026).

Continuous variables were summarized as median and interquartile range, and categorical variables as numbers and percentages. Data were organized and checked in Microsoft Excel. Statistical analyses were performed using IBM SPSS Statistics version 29.0. First-line progression-free survival was assessed by exploratory univariable Cox regression. Associations with second-line treatment were evaluated with Fisher’s exact test and odds ratios with 95% confidence intervals. The Spearman correlation was used to examine the relationship between early marker decline and progression-free survival. Because only a small number of events were observed, no extensive multivariable model was performed. A *p*-value < 0.05 was considered statistically significant. An exploratory threshold of at least 80% tumor marker decline was used to compare observed median first-line PFS between patients with a pronounced early biochemical response and those with a lower decline.

No generative artificial intelligence tool was used for study design, data collection, statistical analysis, interpretation of results, or generation of scientific data. AI-assisted language editing was used only for grammar, clarity, readability, and academic phrasing; the authors reviewed and edited all text and take full responsibility for the manuscript content.

## 3. Results

### 3.1. Baseline Clinical and Pathological Characteristics

The study cohort included 41 patients with germ cell tumors. Baseline clinicopathological characteristics of the study cohort are presented in Table 1. The median age was 37 years. Testicular tumors represented the predominant primary site. Two patients had biopsy-confirmed primary mediastinal germ cell tumors, while three had primary retroperitoneal tumors. Non-seminomatous histology was more common than seminoma.

At the time of analysis, 4 patients (9.8%) had died, while 37 patients (90.2%) remained alive. Median overall survival was not reached.

According to the TNM classification, 4.8% of patients had stage I disease, 46.3% had stage II disease, and 48.7% had stage III disease. Among patients with advanced disease and complete baseline data, 11 were classified as having good-risk disease, 8 as intermediate-risk disease, and 15 as poor-risk disease according to the IGCCCG classification. The IGCCCG risk group could not be determined in five patients because of incomplete baseline data.

Among the 25 patients with non-seminomatous germ cell tumors (NSGCTs), the median age was 36 years. Most NSGCT patients had testicular primary tumors, and embryonal carcinoma was the most frequent histopathological component (Table 2).

### 3.2. Treatment Patterns and First-Line Outcomes

**Figure 1:**
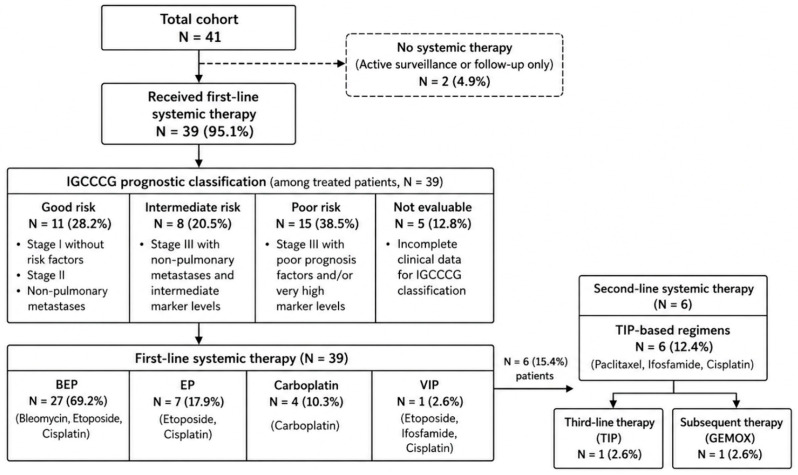
Distribution of patients according to the IGCCCG prognostic classification and systemic treatment pathway.

Among the 41 patients included in the cohort, 39 received first-line systemic therapy. The remaining two patients had localized disease treated with orchiectomy followed by active surveillance with no indication for adjuvant chemotherapy. Among patients treated with first-line systemic therapy, BEP chemotherapy regimen was the most frequently used regimen, followed by EP, carboplatin and VIP. Six patients required second-line systemic treatment. All second-line regimens were TIP. Two patients subsequently received third-line or later systemic treatment. Figure 1 summarizes the distribution of patients according to treatment status and IGCCCG prognostic group, together with the first-line regimens and subsequent systemic therapies administered.

After describing the systemic treatment pathway and the distribution of patients across treatment lines, we further explored clinical and pathological factors associated with first-line progression-free survival (PFS L1) (Table 3).

In exploratory univariable Cox regression analysis, stage III disease and documented metastasis at diagnosis were significantly associated with shorter PFS L1. Stage III disease was associated with a higher risk of progression or need for subsequent treatment. Moreover, documented metastatic disease at diagnosis and a higher percentage of choriocarcinoma component were also associated with shorter PFS L1. Treatment postponement > 7 days during systemic therapy showed a clinically relevant borderline association with shorter PFS L1.

The median orchiectomy-to-treatment interval was 4 weeks. Therefore, when dichotomized according to this median value, an orchiectomy-to-treatment interval > 4 weeks was significantly associated with shorter first-line PFS in exploratory univariable Cox regression analysis (HR 4.20, 95% CI 1.30–13.60; *p* = 0.015).

### 3.3. Early Tumor Marker Decline After the First Chemotherapy Cycle

Early tumor marker decline was evaluable in 16 patients from the overall cohort and in 13 patients from the NSGCT subgroup. We assessed early tumor marker decline as the mean overall percentage decrease in initially elevated tumor markers (Table 4).

A greater mean percentage decline in initially elevated tumor markers after the first chemotherapy cycle was significantly associated with longer first-line PFS in the overall cohort (*p* = 0.019). This association remained significant in the NSGCT subgroup.

Patients with a marker decline of at least 80% had longer observed median first-line PFS compared with those with a lower decline, both in the overall cohort (60.0 vs. 15.0 months; *p* = 0.020) and in the NSGCT subgroup (60.0 vs. 11.0 months; *p* = 0.022). However, when analyzed separately, LDH, AFP, and beta-hCG declines did not reach statistical significance.

### 3.4. Factors Associated with the Need for Second-Line Treatment

Second-line systemic treatment was required in 6 of 41 patients (14.6%). In exploratory univariable analysis, stage III disease was significantly associated with a higher likelihood of requiring second-line treatment compared with stage I/II disease (*p* = 0.013) (Table 5). Documented metastasis at diagnosis showed an even stronger association with second-line treatment requirement (*p* = 0.003).

Baseline tumor marker elevation was significantly associated with subsequent need for second-line therapy. Patients with elevated LDH at diagnosis had a higher likelihood of requiring second-line treatment compared with those with normal LDH (*p* = 0.005). Similar associations were observed for elevated AFP (*p* = 0.013), elevated beta-hCG (*p* < 0.001), and the presence of any elevated baseline tumor marker (*p* = 0.003).

Treatment postponement > 7 days during systemic therapy showed a clinically relevant trend toward a higher need for second-line treatment, although this did not reach statistical significance (*p* = 0.122).

## 4. Discussion

Tumor stage and disease extent at diagnosis remain key determinants of prognosis in patients with testicular cancer [14,19,20,21,22]. In our data, metastasis at diagnosis appeared to capture risk more strongly than advanced stage alone, because tumor dissemination reflects both tumor high volume and aggressive biology. The same variables were also linked to second-line treatment, supporting their use when planning surveillance after first-line therapy.

The baseline marker profile added another prognostic information. Abnormal LDH, AFP, or β-hCG levels at diagnosis were each associated with subsequent treatment escalation, with the strongest estimate observed for β-hCG. It fits with previous work showing that very high hCG values, especially above 50,000 mIU/mL, define a difficult poor-risk subgroup, and that unfavorable marker decline during chemotherapy is associated with poorer outcomes [23,24,25].

Moreover, a greater decline in initially elevated tumor markers was associated with longer first-line PFS in both the overall cohort and the NSGCT subgroup. In our analysis, marker response was expressed as the mean percentage change across all markers elevated at baseline, whereas the GETUG model evaluates the kinetics of AFP and hCG decline after the first chemotherapy cycle. Patients with a marker reduction of at least 80% after cycle 1 had a longer observed median first-line PFS than those with a smaller decline. Although this threshold may reflect an early reduction in viable marker-producing tumor burden, it was used only for exploratory purposes and should not be regarded as a validated clinical decision point. Serum markers remain important for diagnosis, staging, risk assessment, response monitoring, and relapse surveillance [26]. The SEOM-GG guidelines also recommend serial marker assessment during chemotherapy and recognize an inadequate decline as an adverse feature [27]. Similarly, the GETUG-13 strategy used early marker kinetics to identify poor-prognosis patients with an unfavorable response who might benefit from treatment intensification [25]. Further validation is therefore required before the 80% threshold can be applied in clinical practice.

Timing variables require a more careful interpretation. The median interval from orchiectomy to systemic therapy was 4 weeks. In exploratory Cox regression, an orchiectomy-to-treatment interval longer than 4 weeks was associated with shorter PFS.

We also examined treatment postponement of more than seven days as a separate measure of treatment delivery. In our cohort, these delays occurred between planned chemotherapy cycles or treatment sequences and were most often related to treatment-associated toxicity, particularly anemia or neutropenia. Treatment postponement showed a borderline association with shorter first-line PFS and a non-significant trend toward a greater need for second-line therapy. Although maintaining the planned schedule and dose intensity is an important principle in the curative treatment of germ cell tumors, delays may also reflect infection, reduced physiological reserve, treatment toxicity, or more extensive disease. Therefore, treatment postponement may represent both impaired treatment delivery and poorer underlying clinical status. Given the small number of events, wide confidence intervals, and retrospective design, this association should be interpreted cautiously and cannot establish causality.

However, these findings should be interpreted in context. For example, Motzer et al. reported that short chemotherapy-cycle delays of up to 7 days did not reduce complete response or event-free survival in good-risk disseminated germ cell tumors [28]. On the other hand, real world data show that dose delays and reductions occur in routine BEP or EP regimes, even though maintaining dose intensity remains an important treatment principle [29]. Diagnostic delay has also been associated with larger tumors, elevated postoperative markers, metastatic disease, stage III presentation, and greater use of second-line chemotherapy [30]. Taken together, our result is best interpreted as a marker of toxicity, impaired treatment delivery, or a more complex care pathway rather than as proof of causality.

The choriocarcinoma component deserves similar caution. A higher percentage of this component was associated with shorter first-line PFS, but only a few patients had choriocarcinoma elements. Moreover, choriocarcinoma-containing tumors are classically associated with high beta-hCG, distant metastases, and clinically aggressive behavior [31]. The case reported by Jackson et al., involving a major choriocarcinoma component, high hCG, lymphovascular invasion, and pulmonary metastases, illustrates this phenotype [32]. Registry data from Al-Khayal et al. also describe these mixed tumors as uncommon but frequently associated with high-grade histology, distant metastases, and unfavorable outcomes [33].

Several limitations are important for interpreting these results. This was a retrospective study from a single center, with only 41 patients and few progression or second-line treatment events. The marker decline analysis included only patients with available post-cycle 1 marker values. In addition, IGCCCG risk groups were not systematically incorporated into the analysis, which limits direct comparison with established prognostic models. The findings should therefore be considered hypothesis generating.

## 5. Conclusions

In this retrospective cohort of patients with germ cell tumors, poorer first-line outcomes were linked mainly to baseline disease burden. Treatment timing and delivery were also relevant. Therefore, stage III disease and metastasis at diagnosis were associated with shorter first-line PFS, whereas elevated baseline tumor markers were associated with a higher likelihood of second-line systemic therapy. Moreover, early marker decline after the first chemotherapy cycle was associated with longer first-line PFS and may represent a simple marker of treatment sensitivity. Larger multicenter cohorts are needed to validate these observations and to clarify how early biochemical response should be incorporated into risk-adapted follow-up.

## Figures and Tables

**Table 1:** Baseline clinical and pathological characteristics of the overall cohort.

Characteristic	*n* (%)
Age, median, range	37 (29–42), range 23–64
Age ≤ 40 years	30 (73.2%)
Age > 40 years	11 (26.8%)
Cryptorchidism	3 (7.3%)
Current or former smoking	15 (36.6%)
History of varicocele	2 (4.9%)
Family history of testicular cancer	1 (2.4%)
Primary tumor location	
Testis	36 (87.8%)
Retroperitoneum	3 (7.3%)
Anterior mediastinum	2 (4.9%)
Laterality	
Left	19 (50%)
Right	18 (47.4%)
Bilateral	1 (2.6%)
Seminoma	16 (39%)
Non-seminoma	25 (61%)
TNM stage	
Stage I	2 (4.8%)
Stage II	19 (46.3%)
Stage III	20 (48.7%)
Documented metastatic disease at diagnosis	11 (26.8%)
IGCCCG prognostic group	
Good risk	11 (28.2%)
Intermediate risk	8 (20.5%)
Poor risk	15 (38.4%)
Not evaluable	5 (12.8%)
Second-line systemic treatment	6 (14.6%)
Deaths (%)	4 (9.8%)

**Table 2:** Clinical and pathological characteristics of patients with NSGCT.

Characteristic	NSGCT Cohort *n* = 25
Patients, *n*	25
Age, years, median	36 (26–43)
Age ≤ 40 years	18 (72%)
Age > 40 years	7 (28%)
Cryptorchidism	1 (4%)
Current/former smoking	11 (44%)
Varicocele	2 (8%)
Primary tumor location: testis	23 (92%)
Primary tumor location: retroperitoneum	1 (4%)
Primary tumor location: mediastinum	1 (4%)
Embryonal carcinoma component	18 (72%)
Yolk sac tumor component	14 (56%)
Teratoma component	9 (36%)
Choriocarcinoma component	3 (12%)
Documented metastatic disease at diagnosis	7 (28%)
LDH elevated/abnormal at baseline	10 (40%)
AFP elevated/abnormal at baseline	13 (52%)
beta-hCG elevated/abnormal at baseline	11 (44%)
Any tumor marker elevated/abnormal at baseline	14 (56%)
Orchiectomy-to-treatment interval, weeks, median, range	4 (4–7.5), range 3–12

**Table 3:** Exploratory univariable Cox regression analysis evaluating first-line PFS.

Predictor	HR	95% CI	*p*-Value
Stage III vs. stage I/II	11.35	1.31–98.16	0.027
Metastasis at diagnosis	16.71	1.92–145.29	0.011
Age	0.96	0.87–1.06	0.418
Cryptorchidism	2.71	0.32–23.26	0.364
Smoking	0.83	0.15–4.51	0.825
Primary testicular location	0.56	0.06–4.94	0.605
Extragonadal primary location	1.77	0.20–15.48	0.605
Histology: non-seminoma vs. seminoma	3.32	0.39–28.42	0.274
Embryonal carcinoma component	2.58	0.47–14.07	0.275
Yolk sac tumor component	0.98	0.18–5.38	0.986
% choriocarcinoma component	1.03	1.00–1.05	0.036
% embryonal carcinoma component	1.01	0.99–1.03	0.183
Treatment postponement > 7 days	5.27	0.95–29.20	0.057
Orchiectomy-to-treatment > 4 weeks	4.20	1.30–13.60	0.015
LDH decline after C1, %	1.05	0.98–1.12	0.209
AFP decline after C1, %	0.98	0.95–1.01	0.262
beta-hCG decline after C1, %	0.98	0.96–1.01	0.187
Mean early marker decline after C1, %	0.97	0.93–1.01	0.097

**Table 4:** Early tumor marker decline after the first chemotherapy cycle and first-line PFS.

Question	Patients Analyzed	Main Finding	*p*-Value
Does a greater marker decline correlate with longer PFS?	Overall cohort, *n* = 16	Spearman rho = 0.578	0.019
Does this remain true in NSGCT patients?	NSGCT subgroup, *n* = 13	Spearman rho = 0.564	0.045
Do patients with ≥80% decline have longer PFS?	Overall cohort, *n* = 16	Median PFS: 60.0 vs. 15.0 months	0.020
Does the ≥80% threshold remain useful in NSGCT patients?	NSGCT subgroup, *n* = 13	Median PFS: 60.0 vs. 11.0 months	0.022

**Table 5:** Factors associated with the need for second-line systemic treatment.

Predictor	OR	95% CI	*p*-Value
Stage III vs. stage I/II	14.44	1.48–140.79	0.013
Metastasis at diagnosis	24.17	2.37–245.93	0.003
LDH elevated at baseline	20.00	2.00–199.74	0.005
AFP elevated at baseline	14.44	1.48–140.79	0.013
beta-hCG elevated at baseline	42.06	2.14–826.03	<0.001
Any baseline tumor marker elevated	27.70	1.43–534.78	0.003
Treatment postponement > 7 days	7.33	0.88–61.33	0.122
Non-seminoma histology	3.75	0.40–35.54	0.376
Extragonadal primary location	1.55	0.14–16.85	0.567
Smoking	0.85	0.14–5.28	1.000
Cryptorchidism	6.80	0.36–126.91	0.274

## Data Availability

The dataset is not publicly available due to patient confidentiality; anonymized data may be provided upon reasonable request and with appropriate institutional/ethics approval.

## Abbreviations

AFP	alpha-fetoprotein
BEP	bleomycin, etoposide, and cisplatin
β-hCG	beta-human chorionic gonadotropin
C1	first chemotherapy cycle
CI	confidence interval
EP	etoposide and cisplatin
GCT	germ cell tumor
HR	hazard ratio
IGCCCG	International Germ Cell Cancer Collaborative Group
LDH	lactate dehydrogenase
NSGCT	non-seminomatous germ cell tumor
OR	odds ratio
PFS	progression-free survival
VIP	etoposide, ifosfamide, and cisplatin
TIP	paclitaxel, ifosfamide, and cisplatin
